# Previously-initiated hemodialysis as prognostic factor for in-hospital mortality in pneumonia patients with stage 5 chronic kidney disease: Retrospective database study of Japanese hospitals

**DOI:** 10.1371/journal.pone.0213105

**Published:** 2019-02-28

**Authors:** Daisuke Takada, Susumu Kunisawa, Kiyohide Fushimi, Yuichi Imanaka

**Affiliations:** 1 Department of Healthcare Economics and Quality Management, Graduate School of Medicine, Kyoto University, Yoshida Konoe-cho, Sakyo-ku, Kyoto City, Kyoto, Japan; 2 Department of Health Policy and Informatics, Graduate School of Medicine, Tokyo Medical and Dental University, Yushima, Bunkyo-ku, Tokyo, Japan; University of Mississippi Medical Center, UNITED STATES

## Abstract

**Background:**

Some clinicians keep patients in stage 5 chronic kidney disease (CKD) without hemodialysis for a while. This study investigated whether previously-initiated hemodialysis in stage 5 CKD patients may become a prognostic factor for in-hospital mortality due to pneumonia.

**Methods:**

Patient data were obtained from the multi-institutional diagnosis procedure combination database between April 1, 2012 and March 31, 2016. The patients had records of pneumonia as both trigger and major diagnoses and records of end stage renal disease (ESRD) or stage 5 CKD as a comorbidity or other diagnoses on admission and aged 18 years or older. The following factors were adjusted: age, sex, body mass index, Barthel index, orientation disturbance, arterial oxygen saturation, systolic blood pressure, C-reactive protein level or the extent of consolidation on chest radiography, ambulance use, hospitalization within 90 days, and comorbidities upon admission. The primary outcome measure was all-cause in-hospital mortality obtained via multivariable logistic regression analysis using four Models. Model 1 involved complete case analysis with overlapping; one hospitalization per patient was counted as one. Model 2 involved a complete case analysis without overlapping; only the first hospitalization per patient was counted. Model 3 involved multilevel analysis clustered by hospital codes. Model 4 was created after multiple imputation for lacking adjusted factors.

**Results:**

A total of 907 hospitals and 7,726 patients were identified. Hemodialysis was significantly associated with lower in-hospital mortality in all models (odds ratio [OR] = 0.68, 95% confidence interval [CI]: 0.54–0.87 in Model 1; OR = 0.71, 95% CI: 0.55–0.91 in Model 2; OR = 0.67, 95% CI: 0.52–0.86 in Model 3; and OR = 0.68, 95% CI: 0.54–0.87 in Model 4).

**Conclusion:**

Previously-initiated hemodialysis may be an independent prognostic factor for in-hospital mortality in pneumonia patients with end-stage renal disease. This should be borne in mind when considering the time of initiation of dialysis.

## Introduction

End-stage renal disease (ESRD) is one of the leading causes of morbidity and mortality worldwide [1]. The prevalence of ESRD and use of renal replacement therapies (RRTs) such as hemodialysis are expected to increase rapidly, and the number of patients who need RRT will more than double to approximately 5.4 million by 2030 [1]. Some countries, including Japan, have high prevalence of RRT [2]. Furthermore, it is of great concern that the mortality rate due to infection among patients with initiated dialysis has increased year after year [3, 4], and another paper reported that death from pneumonia comprised 46.1% of all infectious disease deaths among dialysis patients in Japan [5].

To reduce infection mortalities among patients with stage 5 chronic kidney disease (CKD), including ESRD, early initiation of dialysis might be beneficial. A well-known randomized controlled trial [6] showed no significant difference in all-cause mortality, infection mortality, and infection hospitalization between two groups: patients whose estimated glomerular filtration rates (eGFRs) were 10.0 to 14.0 mL per minute, which represents the early initiation of hemodialysis, and those with eGFRs between 5.0 to 7.0 mL per minute. However, the conclusion of this trial remains controversial due to sampling bias, because about three-quarters of the screened patients were excluded from the analysis due to physician decision, cancer comorbidity, declined participation, and other reasons.

Pneumonia is a frequently occurring and critical infectious disease, and mortality rates due to pneumonia are higher among patients with ESRD than among the general population [7, 8]. Uremia in patients with ESRD is considered a risk factor, along with several infectious diseases [9, 10], and RRT may reduce mortality in patients with stage 5 CKD. However, studies exploring the association between pneumonia related in-hospital mortality and hemodialysis in stage 5 CKD patients were not found.

This study investigated whether previously-initiated hemodialysis may be a prognostic factor for in-hospital mortality in ESRD patients admitted to acute care hospitals, after adjusting for relevant confounding factors, especially pneumonia severity and daily living activities.

## Methods

### Data source

Briefly, patient data were obtained from the Diagnosis Procedure Combination (DPC) database and our database were acquired from approximately 80% of all the hospitals participating in DPC/Per-Diem Payment System (PDPS) in Japan [11]. The hospitals participating in DPC/PDPS system provided the DPC data to the government for using the payment system. The DPC is a case-mix classification system and the DPC data contains patient details on procedures, medical charges, and clinical summaries. The clinical summaries included various data such as patient age and sex, diagnoses and comorbidities upon admission, and indicators of severity of pneumonia. Diagnosis were coded using the International Classification of Diseases, 10th Revision (ICD-10) codes.

### Inclusion and exclusion criteria of participants

**Fig 1:**
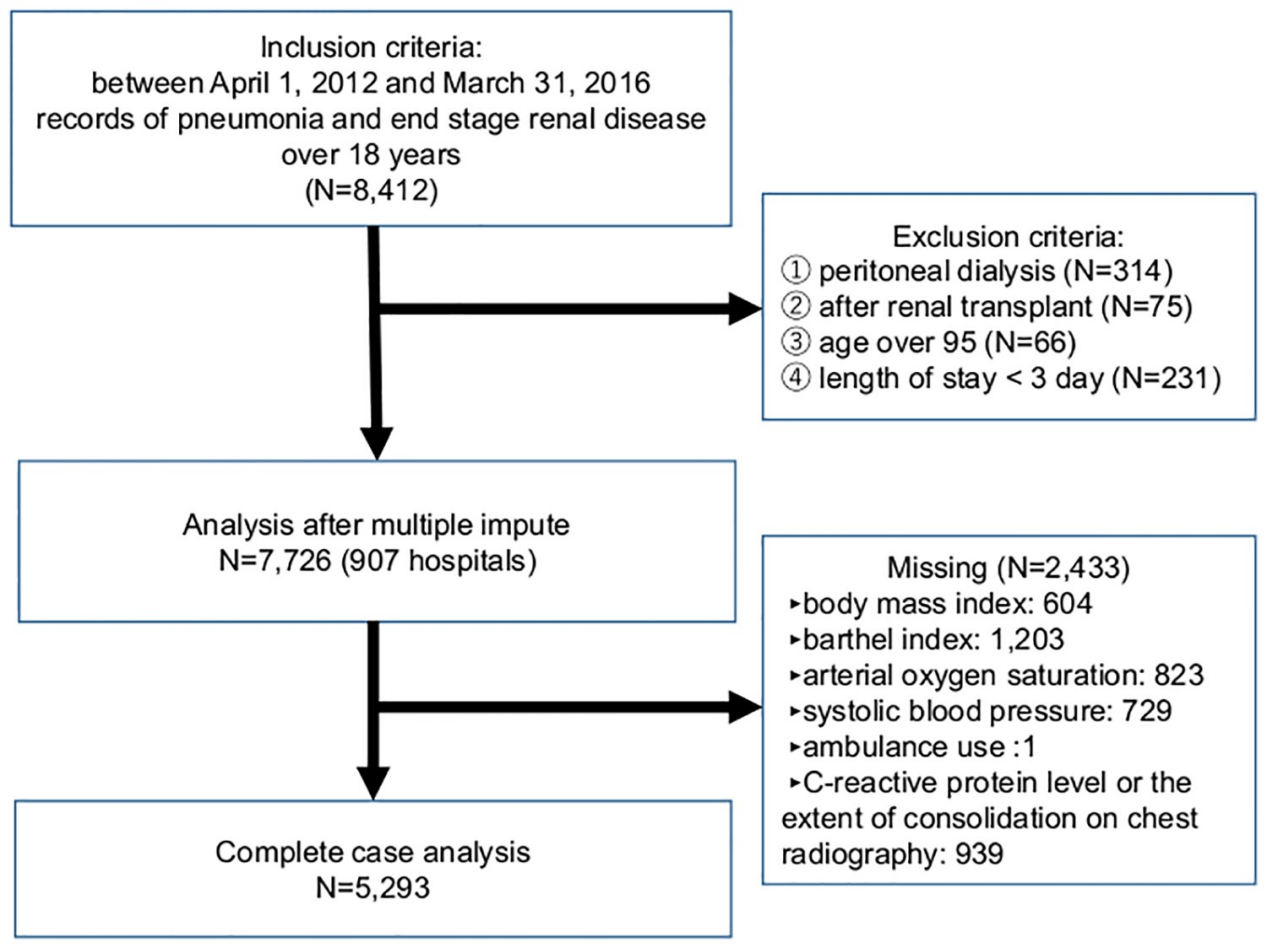
Flow chart of study participant selection.

Fig 1 shows the patient selection process. The inclusion criteria of the present study were as follows: patients with record of pneumonia (J10.0, J11.0, J12–18, A48.1, B01.2, B05.2, B37.1, or B59 in the 2003 version of the ICD-10) in both the trigger and major diagnoses and record of ESRD or stage 5 CKD (N18.0 in the 2003 version of the ICD-10) as a comorbidity or other diagnoses on admission between April 1, 2012 and March 31, 2016, and those aged 18 years or older. However, patients who were on peritoneal dialysis, those who received renal transplant, those older than 95 years, and whose length of stay (LOS) was less than 3 days were excluded from the study.

### Baseline variables

We analyzed patient age, sex, body mass index (BMI), Barthel index, orientation disturbance, arterial oxygen saturation (SpO2), systolic blood pressure [12], C-reactive protein (CRP) level (over 200 mg/L) or the extent of consolidation on chest radiography (≥2/3 of one lung) [13], ambulance use, hospitalization within 90 days at the same hospital, and comorbidities upon admission, diabetes, cancers, heart diseases (congestive heart failure and/or old myocardial infarction), cerebrovascular disease, and liver disease [14]; all covariates were detected on admission. The patients were classified into four groups based on age (<65 years, 65–74 years, 75–84 years, and ≥85 years) and BMI (<17 kg/m^2^, severe or moderate thinness; 17–18.5 kg/m^2^, mild thinness; 18.5–25 kg/m^2^, normal range; and ≥25 kg/m^2^, overweight) according to the guidelines of the World Health Organization [15]. Moreover, the participants were classified into 2 categories based on the Barthel index score [16] (<70 and ≥70), arterial oxygen saturation (<90% and ≥90%), systolic blood pressure (<90 mmHg and ≥90 mmHg) on admission and into 4 groups according to orientation disturbance (Japan Coma Scale score of 0, 1–3, 10–30, and 100–300 [17]). The baseline patient characteristics of the two groups were compared using the *t*-test or chi-squared test or Kruskal-Wallis rank sum test, as appropriate.

### Statistical analyses

The study population was divided into two groups (previously-initiated hemodialysis and not previously-initiated hemodialysis group) based on the claim codes for hemodialysis [18] and diagnosis in order to discriminate temporary or previously introduced hemodialysis. In the present study, the primary outcome measure was all-cause in-hospital mortality obtained via multivariate logistic regression analysis which were adjusted for age, sex, BMI, Barthel index, orientation disturbance, SpO2, systolic blood pressure, CRP level (over 200 mg/L) or the extent of consolidation on chest radiography (≥2/3 of one lung) upon admission, ambulance use, hospitalization within 90 days at the same hospital, and comorbidities upon admission, diabetes, cancers, heart diseases, cerebrovascular disease, and liver disease, as previously stated and categorized in the section regarding baseline variables. In order to ensure the consistency of our results, we analyzed the data using four different models. Model 1 involved complete case analysis with overlapping; one hospitalization per patient was counted as one. Model 2 involved complete case analysis without overlapping; only the first hospitalization per patient was counted. Model 3 involved multilevel analysis clustered by hospital codes. Model 4 involved multiple imputation where the adjusted factors were lacking. We regarded the missing pattern as missing completely at random or missing at random, and we used an extended multiple imputation using the chained equations (MICE) technique [19] that modifies the predictive mean matching method to impute missing data. The results across 20 imputed data sets were combined via averaging, and standard errors were adjusted to reflect both within-imputation variability and between-imputation variability. The imputation procedure uses most of the covariates, including outcomes that may be associated with the missing mechanism to help predict the values for the missing data. The incomplete response variables were BMI, Barthel index, SpO2, systolic blood pressure, CRP level (200 mg/L) or the extent of consolidation on chest radiography (≥2/3 of one lung), and ambulance use. In all logistic regression analyses, we calculated the odds ratios (ORs) and 95% confidence intervals (CIs) for each variable. Additionally, we analyzed propensity scores in three ways, matching, regression adjustment and inverse probability weighting (IPW), to confirm the robustness of results. The propensity score was estimated by multiple logistic regression using all the same variables as Model 1. We performed one-to-one matching 100 times using nearest-neighbor matching without replacement (caliper width < 0.2, standard deviations of the logit of the propensity score) and revealed the 5th, 50th, and 95th percentiles of the estimated risk ratios from the sample average treatment effect for the not previously-initiated hemodialysis group. In the matching and IPW model, we confirmed the balance check with standardized differences.

A two-sided significance level of 0.05 was used, and all analyses were conducted using R version 3.4.1 (The R Development Core Team, Vienna, Austria). Generally, the eGFR of patients on hemodialysis is lower than that of patients who do not receive hemodialysis. Therefore, if the in-hospital mortality rate of patients on hemodialysis was significantly lower than the in-hospital mortality of patients who were not receiving hemodialysis, this difference would exist regardless of a selection bias for eGFR values.

### Sensitivity analysis

In the main analysis, we excluded patients whose LOS was less than 3 days. There were two reasons for choosing 3 days as the cut-off point of LOS. One reason is the suspicion that the hospitalizations were due to pneumonia regardless of the trigger and major diagnoses with pneumonia, because their LOS was shorter. The other reason is that the patients with previously-initiated hemodialysis might have no hemodialysis during hospitalization, and we performed the sensitivity analysis with different cut-off points (1–7 days) in the exclusion criteria.

### Ethical considerations

The study protocol was approved by the ethics committee of Kyoto University Graduate School and Faculty of Medicine (approval number: R0135). This study was conducted in accordance with the ethical guidelines for medical and health research involving human participants issued by the Japanese National Government. These guidelines include a stipulation for the protection of patient anonymity. The data were anonymized, and the requirement for informed consent was waived.

## Results

A total of 907 hospitals and 8,412 patients with ESRD who were admitted due to pneumonia were identified from the DPC database. We excluded patients who were on peritoneal dialysis (n = 314), those who received renal transplant (n = 75), those older than 95 years (n = 66), and whose LOS was less than 3 days (n = 231). Moreover, patients with missing data were not included (n = 2,433). Thus, 5,293 patients were finally included in the complete case analysis (Fig 1). Missing pattern table are shown in S1 Fig.

Table 1 shows the summary of the baseline characteristics, comorbidities, complications, interventions and outcomes in Model 1. Patients in the previously-initiated hemodialysis group were younger and had lower BMI than those in the not previously-initiated hemodialysis group, and the use of ambulance was more frequent in the not previously-initiated hemodialysis group. The in-hospital mortality rate was 15.2% (129 patients) in the not previously-initiated hemodialysis group, and 9.3% (414 patients) in the previously-initiated hemodialysis group. One of the 20 imputed datasets after multiple imputation is shown in S1 Table.

**Table 1:** Patient characteristics, comorbidities, complications, interventions and outcomes in Model 1.

	Not previously-initiated hemodialysis group	Previously-initiated hemodialysis	P value
**Total patient number**	850	4443	
**Patient characteristics**			
** Age, mean (SD)**	77.2 (11.3)	72.6 (10.7)	<0.001
** Age (categorized, %)**			<0.001
**18–64 years (ref.)**	123 (14.5)	1036 (23.3)	
**65–74 years**	197 (23.2)	1550 (34.9)	
**75–84 years**	327 (38.5)	1378 (31.0)	
**85–95 years**	203 (23.9)	479 (10.8)	
** Sex, female (%)**	263 (30.9)	1222 (27.5)	0.045
** Body mass index, mean (SD)**	21.68 (4.27)	20.64 (3.73)	<0.001
** Body mass index (categorized, %)**			<0.001
**Severe, moderate thinness: <17**	94 (11.1)	617 (13.9)	
**Mild thinness: 17–18.5**	103 (12.1)	693 (15.6)	
**Normal (ref.): 18.5–25**	491 (57.8)	2656 (59.8)	
**Pre-obese, obese: over 25**	162 (19.1)	477 (10.7)	
** Arterial oxygen saturation ≥90%(Room Air)**	333 (39.2)	1663 (37.4)	0.355
** Systolic Blood Pressure ≤90**	69 (8.1)	363 (8.2)	1.000
** Orientation disturbance (%)**			<0.001
**JCS: 1–3**	113 (13.3)	527 (11.9)	
**JCS: 10–30**	26 (3.1)	92 (2.1)	
**JCS: 100–300**	17 (2.0)	30 (0.7)	
**JCS: 0 (ref.)**	694 (81.6)	3794 (85.4)	
** Barthel index: poor ≤70**	456 (53.6)	2239 (50.4)	0.089
** CRP level (over 200 mg/L) or the extent of consolidation on chest radiography (≥2/3 of one lung) (%)**	203 (23.9)	1039 (23.4)	0.788
** Ambulance use (%)**	257 (30.2)	1117 (25.1)	0.002
** Recent hospitalization within 90 days (%)**	283 (33.3)	1476 (33.2)	0.999
**Comorbidities**			
** Diabetes (%)**	199 (23.4)	948 (21.3)	0.194
** Cancer (%)**	75 (8.8)	315 (7.1)	0.089
** Heart disease (%)**	234 (27.5)	1059 (23.8)	0.024
** Cerebrovascular (%)**	66 (7.8)	453 (10.2)	0.034
** Liver disease (%)**	6 (0.7)	31 (0.7)	1.000
**Complications and interventions**			
** Sepsis**	6 (0.7)	69 (1.6)	0.079
** Acute respiratory distress syndrome**	5 (0.6)	6 (0.1)	0.025
** Intubations**	32 (3.8)	125 (2.8)	0.165
** Care unit (high or intensive care unit)**	58 (6.8)	283 (6.4)	0.676
**Outcomes**			
** Length of stay [interquartile range]**	15 [9, 25]	13 [9, 20]	<0.001
** In-hospital death (%)**	129 (15.2)	414 (9.3)	<0.001

The baseline patient characteristics of the 2 groups were compared using T-test or chi-squared test or Kruskal-Wallis rank sum test, as appropriate.

Table 2 shows the results of the multivariate analysis of in-hospital mortality after adjusting for the covariates using four models. Hemodialysis was significantly associated with lower in-hospital mortality (OR = 0.69, 95% CI: 0.54–0.88 in Model 1; OR = 0.71, 95% CI: 0.55–0.92 in Model 2; OR = 0.68, 95% CI: 0.52–0.87 in Model 3; and OR = 0.69, 95% CI: 0.54–0.88 in Model 4). Some confounders were associated with high in-hospital mortality: sex, age, severe or moderate thinness, low Barthel index score, low systolic blood pressure, high Japan Coma Scale score, SpO2, hospitalization within 90 days, CRP level (200 mg/L) or the extent of consolidation on chest radiography (≥2/3 of one lung), and cancer and liver disease as comorbidities. The concordance statistics for the scoring system of Model 1 was 0.81 (95% CI: 0.80–0.83).

**Table 2:** Results of the multivariate analysis of in-hospital mortality after adjusting for the covariates using four models. Model 1: complete case analysis with overlap, one hospitalization per patient was counted; Model 2: complete case analysis without overlap, only the first hospitalization per patient was counted; Model 3: multilevel analysis clustered by hospital codes; Model 4: multiple imputation where the adjusted factors were lacking.

	Model 1	Model 2	Model 3	Model 4
**Hemodialysis**	0.69 (0.54 to 0.88)	0.71 (0.55 to 0.92)	0.68 (0.52 to 0.87)	0.69 (0.54 to 0.88)
**Male (reference: female)**	1.31 (1.05 to 1.64)	1.35 (1.06 to 1.71)	1.33 (1.05 to 1.68)	1.31 (1.05 to 1.64)
**Age (reference: 18–64 years)**				
**65–74 years**	2.36 (1.57 to 3.54)	2.26 (1.48 to 3.45)	2.47 (1.63 to 3.75)	2.36 (1.57 to 3.54)
**75–84 years**	4.29 (2.90 to 6.35)	4.12 (2.74 to 6.18)	4.53 (3.03 to 6.77)	4.29 (2.90 to 6.35)
**≥85 years**	4.60 (2.99 to 7.06)	4.35 (2.77 to 6.82)	4.86 (3.13 to 7.54)	4.60 (2.99 to 7.06)
**Body mass index (reference: normal: 18.5–25 kg/m^2^)**				
**Severe, moderate thinness: <17 kg/m^2^**	1.92 (1.49 to 2.46)	1.89 (1.44 to 2.48)	1.98 (1.52 to 2.57)	1.92 (1.49 to 2.46)
**Mild thinness: 17–18.5 kg/m^2^**	0.97 (0.74 to 1.29)	0.97 (0.72 to 1.31)	0.95 (0.72 to 1.27)	0.97 (0.74 to 1.29)
**Pre-obese, obese: ≥25 kg/m^2^**	0.80 (0.54 to 1.17)	0.78 (0.53 to 1.16)	0.80 (0.54 to 1.18)	0.80 (0.54 to 1.17)
**Barthel Index (reference: good **>**70)**				
**Poor ≤70**	2.62 (2.05 to 3.36)	2.64 (2.04 to 3.44)	2.70 (2.09 to 3.48)	2.62 (2.05 to 3.36)
**Arterial oxygen saturation (<90)**	1.99 (1.62 to 2.44)	2.06 (1.65 to 2.57)	2.00 (1.62 to 2.48)	1.99 (1.62 to 2.44)
**Systolic blood pressure (<90)**	3.54 (2.72 to 4.59)	3.27 (2.47 to 4.32)	3.80 (2.87 to 5.03)	3.54 (2.72 to 4.59)
**Japan Coma Scale score (Reference: 0)**				
**1–3**	1.71 (1.34 to 2.20)	1.82 (1.40 to 2.38)	1.74 (1.34 to 2.26)	1.71 (1.34 to 2.20)
**10–30**	1.79 (1.11 to 2.87)	2.17 (1.33 to 3.54)	1.81 (1.11 to 2.97)	1.79 (1.11 to 2.87)
**100–300**	5.81 (3.04 to 11.10)	5.78 (3.01 to 11.11)	6.09 (3.12 to 11.89)	5.81 (3.04 to 11.10)
**Ambulance use**	1.02 (0.82 to 1.27)	0.95 (0.75 to 1.19)	1.03 (0.82 to 1.29)	1.02 (0.82 to 1.27)
**Recent hospitalization (90 days)**	1.29 (1.05 to 1.58)	1.30 (1.04 to 1.62)	1.28 (1.04 to 1.58)	1.29 (1.05 to 1.58)
**CRP 200 mg/L or extent of consolidation on chest X-ray ≥2/3 of one lung**	1.87 (1.52 to 2.32)	1.85 (1.48 to 2.32)	1.94 (1.56 to 2.42)	1.87 (1.52 to 2.32)
**Diabetes**	0.81 (0.62 to 1.05)	0.80 (0.61 to 1.05)	0.80 (0.61 to 1.04)	0.81 (0.62 to 1.05)
**Cancer**	1.59 (1.14 to 2.21)	1.56 (1.09 to 2.23)	1.61 (1.15 to 2.26)	1.59 (1.14 to 2.21)
**Heart disease**	1.01 (0.81 to 1.26)	0.94 (0.74 to 1.19)	1.02 (0.81 to 1.29)	1.01 (0.81 to 1.26)
**Cerebrovascular disease**	0.62 (0.43 to 0.88)	0.63 (0.43 to 0.92)	0.61 (0.43 to 0.88)	0.62 (0.43 to 0.88)
**Liver disease**	3.15 (1.28 to 7.73)	3.04 (1.23 to 7.53)	3.39 (1.33 to 8.62)	3.15 (1.28 to 7.74)

Odds ratio (95% confidence interval)

The results of propensity score analysis: the median estimated relative risk was 0.71 (5th and 95th percentiles: 0.65 to 0.79) in matching, the OR was 0.71 (95% CI: 0.57–0.89) in regression adjustment, 0.77 (95% CI: 0.61–0.98) in IPW. The results using propensity score were similar to our main analysis. Balance check with standardized differences before and after propensity score analysis are shown in S2 Table.

As shown in S3 Table, the sensitivity analysis showed little difference among the point estimates of the ORs.

## Discussion

This multicenter study showed that previously initiated hemodialysis was associated with low in-hospital mortality among patients with stage 5 CKD who were admitted due to pneumonia, after adjusting for relevant confounding factors such as the severity of pneumonia and activities of daily living. The good effect of hemodialysis was similar among the four models.

Each model contributed to the consistency of the effect on hemodialysis among pneumonia patients. When only the first admission for a patient was selected, we lost information regarding the worst admission event for the same patient. We then analyzed all the admissions for the same patient (Model 2). Mass infections, due to the close distance between patients during treatment in hemodialysis facilities, may influence the effect of hemodialysis; thus, we performed multilevel logistic regression analysis (Model 3). Moreover, the lack of data may introduce selection bias; we, therefore, used multiple imputation (Model 4). Missing data might have influence on the comparison; 32% of patients had missing data, and we analyzed the missing pattern as missing completely at random or missing at random. If the missing pattern were missing not at random, these missing data might have influence on the comparison. However, each model revealed a similar effect of hemodialysis on in-hospital mortality.

In our analysis, the severity of pneumonia was adjusted rigorously. One of the most reliable scoring systems for confirming the severity of pneumonia is the CURB-65 scale, which was adopted from an earlier version developed by the British Thoracic Society [20]. The A-DROP scoring system, which is a modified version of the CURB-65 system developed by the Japanese Respiratory Society, has a higher level of discrimination than the CURB-65 scale [12]. The A-DROP scoring system comprises five variables: age ≥ 70 years (male) or ≥ 75 years (female); dehydration is defined as blood urea nitrogen concentration ≥ 7.5 mmol/L or clinical condition; the respiratory condition is confirmed by arterial oxygen saturation ≤ 90%; orientation disturbance; and systolic blood pressure ≤ 90 mmHg. Blood urea nitrogen seems to make no sense because patients with stage 5 CKD had higher blood urea nitrogen and we analyzed the other variables, and almost all the results of other variables were similar to those reported in previous studies [14].

Severe or moderate thinness (BMI below 17 kg/m^2^) was significantly associated with increased OR for mortality relative to the normal (BMI: 18.5–25 kg/m^2^). In contrast, pre-obese condition or obesity (BMI over 25 kg/m^2^) was associated with decreased mortality. However, the results were not significant. This finding was similar to those of recent studies [21]. Interestingly, mild thinness (17–18.5 kg/m^2^) was not correlated with in-hospital mortality (OR = 0.97, 95% CI: 0.74–1.29). However, severe or moderate thinness (BMI below 17 kg/m^2^) was significantly associated with high in-hospital mortality (OR = 1.92, 95% CI: 1.49–2.46) in our analysis.

In our study, the good effects of hemodialysis on in-hospital mortality among stage 5 CKD patients, including those with ESRD, are attributed to several hypotheses. The first hypothesis is that hemodialysis may improve the uremia in stage 5 CKD patients [22, 23]. Patients with concurrent infection and uremia who are on hemodialysis have increased levels of serum inflammatory factors. Those who are continuously undergoing high throughput blood purification have decreased serum levels of CRP, interleukin-2, and tumor necrosis factor-α, and high throughput hemodialysis may be beneficial for the prevention of infections in patients with uremia. Thus, ESRD-associated inflammation is due to the activation of the immune system, and RRT might improve the immune system.

The second hypothesis involves the early detection and treatment of pneumonia among hemodialysis patients, because hemodialysis patients attend hospital more frequently than the not previously-initiated hemodialysis group. The Japanese Society for Dialysis Therapy (JSDT) guidelines recommended hemodialysis three times per week. The grade for the strength of recommendation is 1B (strong recommendation) [24]. Hemodialysis patients may immediately be assessed and treated by medical staffs if they felt even slightly ill. In contrast, patients who are not on hemodialysis would come to the hospital only when their physical condition is extremely worse. Before our analysis (Table 1), the use of ambulance among patients not on hemodialysis was significantly more frequent than that among patients on hemodialysis, and logistic regression analysis was performed after adjusting for ambulance use.

The third hypothesis is malnutrition due to decreased dietary protein intake. The National Kidney Foundation Kidney Disease Outcomes Quality Initiative guidelines on hypertension and antihypertensive agents in 2004 and 2007 recommended a reduction in dietary protein intake (approximately 0.6–0.8 g/kg body weight per day) [25]. Moreover, based on the Japanese Society of Nephrology guidelines 2009 and 2013, dietary protein restriction has been considered to control the progression of renal dysfunction in patients with chronic kidney disease stages 3–5 [26]. Multiple logistic regression analysis was conducted after adjusting for BMI. However, we could not consider the nutrition status of the patients, such as those with sarcopenic obesity [27].

Previous studies have reported that hemodialysis in patients with stage 5 CKD or ESRD, considering the early initiation of dialysis, had no significant effect on the rate of death from infectious events. In the Initiating Dialysis Early and Late (IDEAL) study, Cooper et al. [6] reported that the hazard ratio of death from infection in patients with early initiation of hemodialysis was not significant, which is different from our results. This result may be attributed to several hypotheses. One hypothesis is that their protocol only confirmed the reduced urea ratio for the measurement of dialysis adequacy. However, based on the JSDT guidelines, a decreased urea level along with dialysis time is recommended [24]. Consequently, it may lead to a higher in-hospital mortality rate among hemodialysis patients because of early detection and treatment. Another hypothesis is that multidrug-resistant Gram-negative bacteria may be found in hemodialysis facilities [28]. Thus, we conducted multilevel analysis in Model 3, and the result did not change.

The present study has key strengths. It has a large statistical power in multiple facilities. Second, our analyses were adjusted for the severity of pneumonia and activities of daily living, which were considered to be risk factors for in-hospital mortality. Third, because of the healthcare infrastructure of Japan, patients have long enjoyed universal access to dialysis with a cost of approximately $100 per month, and the income and revenue were unlikely to become confounding factors. These three strengths were why the concordance statistic was high regardless of the high risk of death among the included patients with stage 5 CKD. Finally, the consistency of the OR was confirmed according to the four models analyzed.

### Limitations

Our study has some limitations. First, the DPC administrative database does not provide detailed clinical information, including the medical history or laboratory findings, such as eGFR. However, if we could measure the eGFR, the eGFR in previously introduced hemodialysis patients would be lower than that without hemodialysis; this selection bias may lead to reduce the difference. Therefore, our conclusion will not be affected by this bias. Moreover, the explanatory variables were based on claims data and diagnosis, the incidence of comorbidities among pneumonia cases identified in this study may be lower than the actual incidence.

Second, unmeasured confounders may exist because of the retrospective nature of the study, such as pneumococcal vaccine, the existence of drug-resistant bacteria, hesitation about introducing hemodialysis. Some biological markers, such as indoxyl sulfate, p-cresyl sulfate and hippurate, secreted by the proximal tubular cells of the kidney have been associated with progression of CKD and may be linked to fatigue, uremic pruritus, anorexia, and other component symptoms of the uremic syndrome [29]. Whether hemodialysis improves the in-hospital mortality rate is not concluded, but our analyses imply that previously-initiated hemodialysis may be a prognostic factor for in-hospital mortality in ESRD patients in a practical setting.

Third, this analysis only included the Japanese population in the Japanese healthcare system. These findings might not be generalizable to other countries.

## Conclusion

In conclusion, our findings suggest that previously-initiated hemodialysis is an independent prognostic factor for in-hospital mortality in pneumonia patients with ESRD. This should be borne in mind when considering the time when dialysis was initiated. There is also a need for further studies; if possible, randomized control trials with reduced selection bias should be conducted to explore it further.

## Supporting information

S1 Fig Missing pattern table. (TIF)

S1 Table One of the imputed datasets. (DOCX)

S2 Table Balance check with standardized differences before and after propensity score analysis. (DOCX)

S3 Table Sensitivity analysis for length of stay in exclusion criteria. (DOCX)
