# Supplementary figures and images for: Previously-initiated hemodialysis as prognostic factor for in-hospital mortality in pneumonia patients with stage 5 chronic kidney disease: Retrospective database study of Japanese hospitals
Source: PLoS One. 2019 Feb 28;14(2):e0213105. doi: 10.1371/journal.pone.0213105 (PMC6394945; doi:10.1371/journal.pone.0213105)

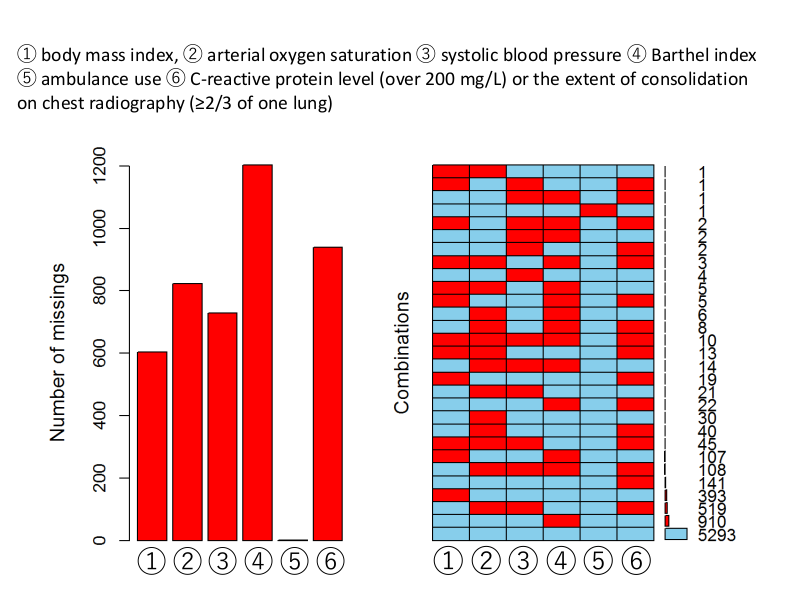

Supplement: S1 Fig — (TIF) [file pone.0213105.s001.tif]
