# Supplementary material for: Previously-initiated hemodialysis as prognostic factor for in-hospital mortality in pneumonia patients with stage 5 chronic kidney disease: Retrospective database study of Japanese hospitals
Source: PLoS One. 2019 Feb 28;14(2):e0213105. doi: 10.1371/journal.pone.0213105 (PMC6394945; doi:10.1371/journal.pone.0213105)
Supplement: S1 Table — (DOCX) [file pone.0213105.s002.docx]

Supplementary Table 1. One of the imputed datasets

|  | Not previously-initiated hemodialysis group | Previously-initiated hemodialysis | P value |
| --- | --- | --- | --- |
| Total patient number | 1232 | 6494 |  |
| Age (categorized , %) |  |  | <0.001 |
| 18-64 years(ref.) | 167 (13.6) | 1444 (22.2) |  |
| 65-74 years | 271 (22.0) | 2171 (33.4) |  |
| 75-84 years | 484 (39.3) | 2151 (33.1) |  |
| 85-95 years | 310 (25.2) | 728 (11.2) |  |
| Sex, female (%) | 389 (31.6) | 1839 (28.3) | 0.023 |
| Body mass index (categorized , %) |  |  | <0.001 |
| Severe, moderate thinness: <17 | 137 (11.1) | 947 (14.6) |  |
| Mild thinness: 17-18.5 | 160 (13.0) | 981 (15.1) |  |
| Normal (ref.): 18.5-25 | 716 (58.1) | 3853 (59.3) |  |
| Pre-obese, obese: over 25 | 219 (17.8) | 713 (11.0) |  |
| Arterial oxygen saturation ≥90%(Room Air) | 502 (40.7) | 2534 (39.0) | 0.269 |
| Systolic Blood Pressure ≤90 | 111 ( 9.0) | 552 ( 8.5) | 0.596 |
| Orientation disturbance (%) |  |  | <0.001 |
| JCS: 1-3 | 186 (15.1) | 784 (12.1) |  |
| JCS: 10-30 | 41 ( 3.3) | 149 ( 2.3) |  |
| JCS: 100-300 | 24 ( 1.9) | 57 ( 0.9) |  |
| JCS: 0 (ref.) | 981 (79.6) | 5504 (84.8) |  |
| Barthel index: poor ≤70 | 705 (57.2) | 3359 (51.7) | <0.001 |
| CRP level (over 200 mg/L) or the extent of consolidation on chest radiography (≥2/3 of one lung) (%) | 312 (25.3) | 1551 (23.9) | 0.295 |
| Ambulance use (%) | 395 (32.1) | 1729 (26.6) | <0.001 |
| Recent hospitalization within 90 days (%) | 401 (32.5) | 2124 (32.7) | 0.94 |
| Diabetes (%) | 272 (22.1) | 1386 (21.3) | 0.59 |
| Cancer (%) | 107 ( 8.7) | 446 ( 6.9) | 0.027 |
| Heart disease (%) | 347 (28.2) | 1516 (23.3) | <0.001 |
| Cerebrovascular (%) | 95 ( 7.7) | 654 (10.1) | 0.012 |
| Liver disease (%) | 7 ( 0.6) | 42 ( 0.6) | 0.902 |
| In-hospital death (%) | 1002/230 (81.3/18.7) | 5830/664 (89.8/10.2) | <0.001 |
| The baseline patient characteristics of the 2 groups were compared using the T test or chi-squared test, as appropriate. | | | |
