# Supplementary material for: Previously-initiated hemodialysis as prognostic factor for in-hospital mortality in pneumonia patients with stage 5 chronic kidney disease: Retrospective database study of Japanese hospitals
Source: PLoS One. 2019 Feb 28;14(2):e0213105. doi: 10.1371/journal.pone.0213105 (PMC6394945; doi:10.1371/journal.pone.0213105)
Supplement: S2 Table — (DOCX) [file pone.0213105.s003.docx]

Supplementary Table 2. Balance check with standardized differences before and after propensity score analysis

|  | Crude | | | Matching | | | Inverse probability weighting | | |
| --- | --- | --- | --- | --- | --- | --- | --- | --- | --- |
|  | Not previously-initiated | Previously-initiated | SMD | Not previously-initiated | Previously-initiated | SMD | Not previously-initiated | Previously-initiated | SMD |
| Total patient number | 850 | 4443 |  | 844 | 844 |  | 5271.21 | 5296.12 |  |
| Age (mean (SD)) | 77.16 (11.33) | 72.63 (10.73) | 0.41 | 77.08 (11.33) | 76.26 (11.07) | 0.073 | 73.38 (11.89) | 73.29 (10.83) | 0.008 |
| Age (categorized, %) |  |  | 0.463 |  |  | 0.05 |  |  | 0.015 |
| 18-64 years (ref.) | 123 (14.5) | 1036 (23.3) |  | 123 (14.6) | 133 (15.8) |  | 1169.1 (22.2) | 1159.1 (21.9) |  |
| 65-74 years | 197 (23.2) | 1550 (34.9) |  | 197 (23.3) | 184 (21.8) |  | 1702.0 (32.3) | 1745.6 (33.0) |  |
| 75-84 years | 327 (38.5) | 1378 (31.0) |  | 326 (38.6) | 335 (39.7) |  | 1722.6 (32.7) | 1707.7 (32.2) |  |
| 85-95 years | 203 (23.9) | 479 (10.8) |  | 198 (23.5) | 192 (22.7) |  | 677.5 (12.9) | 683.7 (12.9) |  |
| Sex, female (%) | 263 (30.9) | 1222 (27.5) | 0.076 | 259 (30.7) | 263 (31.2) | 0.01 | 1391.0 (26.4) | 1479.4 (27.9) | 0.035 |
| Body mass index, mean (SD) | 21.68 (4.27) | 20.64 (3.73) | 0.259 | 21.66 (4.26) | 21.41 (4.09) | 0.06 | 21.00 (4.04) | 20.77 (3.81) | 0.059 |
| Body mass index (categorized, %) |  |  | 0.252 |  |  | 0.027 |  |  | 0.034 |
| Severe, moderate thinness: <17 | 94 (11.1) | 617 (13.9) |  | 94 (11.1) | 91 (10.8) |  | 763.7 (14.5) | 712.7 (13.5) |  |
| Mild thinness: 17-18.5 | 103 (12.1) | 693 (15.6) |  | 102 (12.1) | 104 (12.3) |  | 754.7 (14.3) | 795.8 (15.0) |  |
| Normal (ref.): 18.5-25 | 491 (57.8) | 2656 (59.8) |  | 491 (58.2) | 484 (57.3) |  | 3102.3 (58.9) | 3143.7 (59.4) |  |
| Pre-obese, obese: over 25 | 162 (19.1) | 477 (10.7) |  | 157 (18.6) | 165 (19.5) |  | 650.5 (12.3) | 644.0 (12.2) |  |
| Arterial oxygen saturation ≥ 90% (room air) | 333 (39.2) | 1663 (37.4) | 0.036 | 329 (39.0) | 342 (40.5) | 0.031 | 1920.2 (36.4) | 1994.7 (37.7) | 0.026 |
| Systolic Blood Pressure ≤90 | 69 ( 8.1) | 363 ( 8.2) | 0.002 | 69 ( 8.2) | 67 ( 7.9) | 0.009 | 459.7 ( 8.7) | 434.5 ( 8.2) | 0.019 |
| Orientation disturbance (%) |  |  | 0.143 |  |  | 0.049 |  |  | 0.015 |
| JCS: 1-3 | 113 (13.3) | 527 (11.9) |  | 112 (13.3) | 102 (12.1) |  | 627.3 (11.9) | 642.1 (12.1) |  |
| JCS: 10-30 | 26 ( 3.1) | 92 ( 2.1) |  | 26 ( 3.1) | 22 ( 2.6) |  | 106.3 ( 2.0) | 116.5 ( 2.2) |  |
| JCS: 100-300 | 17 ( 2.0) | 30 ( 0.7) |  | 15 ( 1.8) | 14 ( 1.7) |  | 50.9 ( 1.0) | 50.4 ( 1.0) |  |
| JCS: 0 (ref.) | 694 (81.6) | 3794 (85.4) |  | 691 (81.9) | 706 (83.6) |  | 4486.7 (85.1) | 4487.1 (84.7) |  |
| Barthel index: poor ≤70 | 456 (53.6) | 2239 (50.4) | 0.065 | 450 (53.3) | 459 (54.4) | 0.021 | 2671.3 (50.7) | 2698.5 (51.0) | 0.005 |
| CRP level (over 200 mg/L) or the extent of consolidation on chest radiography (≥2/3 of one lung) (%) | 203 (23.9) | 1039 (23.4) | 0.012 | 203 (24.1) | 192 (22.7) | 0.031 | 1215.7 (23.1) | 1246.7 (23.5) | 0.011 |
| Ambulance use (%) | 257 (30.2) | 1117 (25.1) | 0.114 | 253 (30.0) | 244 (28.9) | 0.023 | 1358.6 (25.8) | 1377.1 (26.0) | 0.005 |
| Recent hospitalization within 90 days (%) | 283 (33.3) | 1476 (33.2) | 0.002 | 282 (33.4) | 291 (34.5) | 0.023 | 1801.4 (34.2) | 1758.6 (33.2) | 0.02 |
| Diabetes (%) | 199 (23.4) | 948 (21.3) | 0.05 | 197 (23.3) | 201 (23.8) | 0.011 | 1190.3 (22.6) | 1148.0 (21.7) | 0.022 |
| Cancer (%) | 75 ( 8.8) | 315 ( 7.1) | 0.064 | 75 ( 8.9) | 70 ( 8.3) | 0.021 | 408.5 ( 7.8) | 392.8 ( 7.4) | 0.013 |
| Heart disease (%) | 234 (27.5) | 1059 (23.8) | 0.085 | 230 (27.3) | 240 (28.4) | 0.026 | 1294.1 (24.6) | 1294.2 (24.4) | 0.003 |
| Cerebrovascular (%) | 66 ( 7.8) | 453 (10.2) | 0.085 | 66 ( 7.8) | 61 ( 7.2) | 0.022 | 460.5 ( 8.7) | 518.4 ( 9.8) | 0.036 |
| Liver disease (%) | 6 ( 0.7) | 31 ( 0.7) | 0.001 | 6 ( 0.7) | 7 ( 0.8) | 0.014 | 43.3 ( 0.8) | 37.5 ( 0.7) | 0.013 |
| Abbreviation: SMD, standardized mean difference | | | | | | | | | |
