# Supplementary material for: Previously-initiated hemodialysis as prognostic factor for in-hospital mortality in pneumonia patients with stage 5 chronic kidney disease: Retrospective database study of Japanese hospitals
Source: PLoS One. 2019 Feb 28;14(2):e0213105. doi: 10.1371/journal.pone.0213105 (PMC6394945; doi:10.1371/journal.pone.0213105)
Supplement: S3 Table — (DOCX) [file pone.0213105.s004.docx]

Supplementary Table 3. Sensitivity analysis for length of stay in exclusion criteria

| Exclusion criteria  Less than the number (length of stay) | Sample size | Odds ratio (95% confidence interval) |
| --- | --- | --- |
| 1 | 5427 | 0.55 (0.44 to 0.68) |
| 2 | 5392 | 0.57 (0.46 to 0.72) |
| 3 | 5293 | 0.69 (0.54 to 0.88) |
| 4 | 5196 | 0.75 (0.58 to 0.97) |
| 5 | 5071 | 0.83 (0.64 to 1.10) |
| 6 | 4916 | 0.82 (0.62 to 1.08) |
| 7 | 4703 | 0.83 (0.62 to 1.10) |
| 8 | 4480 | 0.86 (0.64 to 1.15) |
